# Limited Adipogenic Differentiation Potential of Human Dental Pulp Stem Cells Compared to Human Bone Marrow Stem Cells

**DOI:** 10.3390/ijms252011105

**Published:** 2024-10-16

**Authors:** Isaac Maximiliano Bugueno, Giuseppe Alastra, Anamaria Balic, Bernd Stadlinger, Thimios A. Mitsiadis

**Affiliations:** 1 Orofacial Development and Regeneration, Institute of Oral Biology, Faculty of Medicine, Centre of Dental Medicine, University of Zurich, CH-8032 Zurich, Switzerland; isaac.buguenovaldebenito@zzm.uzh.ch (I.M.B.); giuseppe.alastra2@unibo.it (G.A.); anamaria.balic@zzm.uzh.ch (A.B.); 2 Department of Veterinary Medical Sciences, University of Bologna, 40126 Bologna, Italy; 3 Clinic of Cranio-Maxillofacial and Oral Surgery, University of Zurich, CH-8032 Zurich, Switzerland; bernd.stadlinger@zzm.uzh.ch

**Keywords:** adipogenic potential, bone marrow stem cells, dental pulp stem cells, human, differentiation, lipid vesicles, SWAT cells, WNT signalling, NOTCH signalling

## Abstract

Bone marrow and teeth contain mesenchymal stem cells (MSCs) that could be used for cell-based regenerative therapies. MSCs from these two tissues represent heterogeneous cell populations with varying degrees of lineage commitment. Although human bone marrow stem cells (hBMSCs) and human dental pulp stem cells (hDPSCs) have been extensively studied, it is not yet fully defined if their adipogenic potential differs. Therefore, in this study, we compared the in vitro adipogenic differentiation potential of hDPSCs and hBMSCs. Both cell populations were cultured in adipogenic differentiation media, followed by specific lipid droplet staining to visualise cytodifferentiation. The in vitro differentiation assays were complemented with the expression of specific genes for adipogenesis and osteogenesis–dentinogenesis, as well as for genes involved in the Wnt and Notch signalling pathways. Our findings showed that hBMSCs formed adipocytes containing numerous and large lipid vesicles. In contrast to hBMSCs, hDPSCs did not acquire the typical adipocyte morphology and formed fewer lipid droplets of small size. Regarding the gene expression, cultured hBMSCs upregulated the expression of adipogenic-specific genes (e.g., *PPARγ2*, *LPL*, *ADIPONECTIN*). Furthermore, in these cells most Wnt pathway genes were downregulated, while the expression of NOTCH pathway genes (e.g., *NOTCH1*, *NOTCH3*, *JAGGED1*, *HES5*, *HEY2*) was upregulated. hDPSCs retained their osteogenic/dentinogenic molecular profile (e.g., *RUNX2*, *ALP*, *COLIA1*) and upregulated the WNT-specific genes but not the NOTCH pathway genes. Taken together, our in vitro findings demonstrate that hDPSCs are not entirely committed to the adipogenic fate, in contrast to the hBMSCs, which are more effective to fully differentiate into adipocytes.

## 1. Introduction

Mesenchymal stem cells (MSCs) are essential components of tissue homeostasis, repair, and regeneration throughout life [1,2]. MSCs are multipotent, clonogenic cells that can be easily manipulated and expanded in vitro [1]. In response to lineage-selective inducers, a cooperative action between specific transcription factors and signalling molecules, MSCs can generate cells of distinctive mesodermal lineages, including osteoblasts and adipocytes [1,2]. However, studies have shown that inducers of one particular cell lineage (e.g., adipogenic) often inhibit differentiation along another cell lineage (e.g., osteogenic) [3,4,5,6,7,8]. For example, the transcription factor peroxisome proliferator-activated receptor gamma (PPARγ) induces adipogenesis while inhibiting osteogenesis [6]. In contrast, the function of PPARγ is suppressed in MSCs by osteogenic inducers, such as molecules of the canonical Wnt signalling pathway [3,8]. In recent years, a variety of MSC populations have started to be used in clinics in order to support and enhance the regenerative potential of damaged tissues [9,10,11].

Cells from the bone marrow were the first source of MSCs used in clinics [12], and human bone marrow stem cells (hBMSCs) remain the gold standard for the analysis of MSC properties and functions [13]. In more recent years, specific populations of MSCs were discovered in almost every tissue and organ of the human body [12,13,14,15,16]. In teeth, MSCs were first isolated from the human dental pulp tissue and were accordingly named dental pulp stem cells (hDPSCs) [17,18,19]. Subsequent studies have revealed the presence of other specific dental tissues, including the MSCs in periodontal ligament [20], apical papilla [21], and dental follicle [22]. Extensive studies on hDPSCs have shown that these cells have a remarkable ability to differentiate into various cell lines (e.g., osteoblastic, odontoblastic, chondroblastic, neurogenic, adipogenic) [23,24], and they possess immunosuppressive activity [25]. Because of these properties, hDPSCs hold the potential for clinical application and constitute a very appealing alternative to hBMSCs for regenerative treatments due to their easy accessibility and immediate availability [26]. The potential use of hDPSCs for bone regeneration has been examined in vivo, and some clinical investigations have even demonstrated their ability to restore pulp tissue after pulpectomy [7,27,28,29,30]. Additionally, some solid studies on the differentiation potential of hDPSCs indicate their commitment to form odontoblasts or osteoblasts, but these potentials were not always quantitatively analysed in all studies. This is particularly true for their adipogenic potential, where the findings from several other studies vary greatly and are even contradictory or inconclusive [22,23].

Bone marrow and teeth have different embryonic origins and exhibit distinctive transcriptional profile signatures [31,32,33], and, therefore, it is likely that the adipogenic differentiation potentials of hBMSCs and hDPSCs are not identical. However, the commitment of hBMSCs and hDPSCs towards the adipogenic lineage has never been thoroughly examined in a comparative way. This study is a first comprehensive and quantitative comparative analysis of adipogenic differentiation potential of MSCs from human dental pulp and bone marrow. It highlights distinct differences in the lineage commitment between MSC populations isolated from these two organs, that might influence the outcome of the regenerative therapies.

## 2. Results

### 2.1. Reduced Adipogenic Differentiation of hDPSCs When Compared to hBMSCs

**Figure 1:**
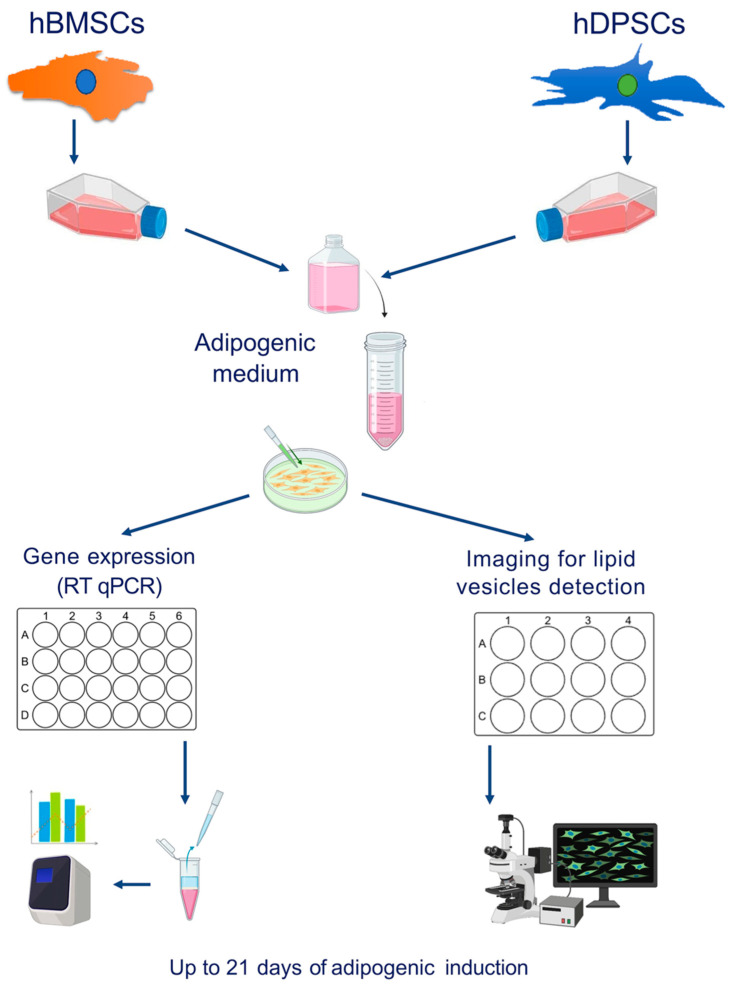
**Experimental set-up of adipogenic induction in hBMSCs and hDPSCs.** hDPSCs and hBMSCs were cultured in 2D (6-well plates, 24-well plates, and μ-slide 4-well uncoated plates), and three time points were chosen for gene expression by RT qPCR (0, 7, and 21 days of induction) and six time points for lipid vesicle analysis (0, 7, 10, 14, and 21 days of induction). Bright-field and confocal scanning microscopy analyses before and after specific staining of lipid droplets (Nile red and LipidSpot^TM^) were performed up to 21 days of adipogenic induction.

**Figure 2:**
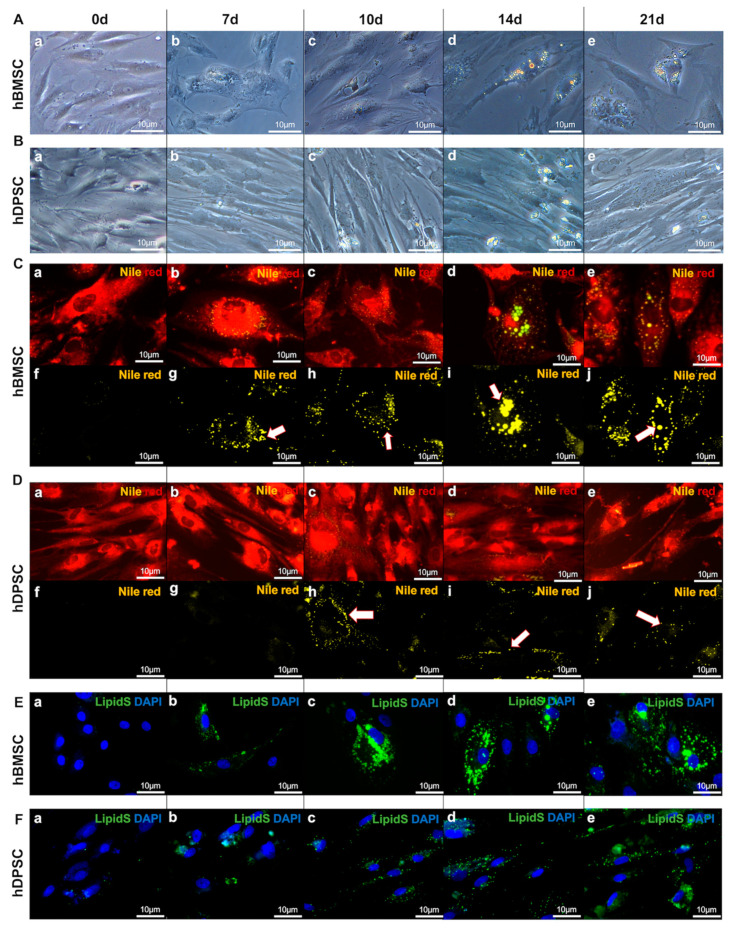
**Adipogenic differentiation of hBMSCs and hDPSCs.** (**A**) Inverted bright-field microscopy on cells at 0 (a), 7 (b), 10 (c), 14 (d), and 21 (e) days of adipogenic induction in hBMSCs. (**B**) Inverted bright-field microscopy on cells at 0 (a), 7 (b), 10 (c), 14 (d), and 21 (e) days of adipogenic induction in hDPSCs. (**C**) Wide-field inverted fluorescence microscopy of hBMSCs at 0 (a,f), 7 (b,g), 10 (c,h), 14 (d,i), and 21 (e,j) days of differentiation (in yellow and red: Nile red staining). All scale bars represent 10 μm; n = 6. Below each photo, the isolated yellow channel allows better visualisation of the lipid droplets, indicated by the white arrows. (**D**) Wide-field inverted fluorescence microscopy of hDPSCs at 0 (a,f), 7 (b,g), 10 (c,h), 14 (d,i), and 21 (e,j) days of differentiation (in yellow and red: Nile red staining). All scale bars represent 10 μm; n = 6. Below each photo, the yellow channel allows better visualisation of the lipid droplets, indicated by the white arrows. (**E**,**F**) Confocal high-speed multispectral spinning-disk microscopy of lipid droplet staining of hBMSCs and hDPSCs at 0 (a), 7 (b), 10 (c), 14 (d), and 21 (e) days of differentiation (green colour: LipidSpot^TM^ 488 staining; blue colour: DAPI staining). Scale bars: 10 μm.

We cultured hDPSCs and hBMSCs until they reached confluence, which is the time point that their differentiation process into adipocytes was induced (Figure 1). hBMSCs cultured in adipogenic medium for 3 days started to acquire the characteristic morphology of adipocytes, and the number of these differentiating cells was constantly increased in the following days (Figure 2A). By contrast, hDPSCs cultured in adipogenic conditions displayed subtle and non-specific changes in their overall appearance, except for a few cells that acquired the round shape of adipocytes (Figure 2B). These hDPSC-originated adipocytes appeared around 7 days post-induction, but the number of did not significantly increase in the following days (Figure 2B).

### 2.2. Comparison of the Lipid Vesicle Formation between the hBMSC- and hDPSC-Originated Adipocytes

**Figure 3:**
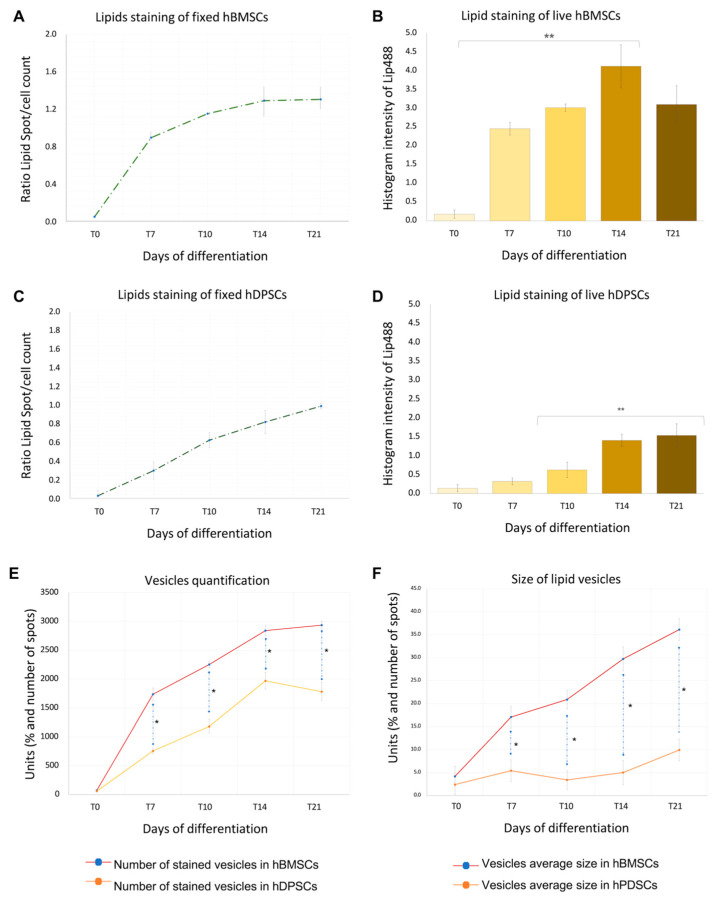
**Comparison of the lipid vesicle formation between the hBMSC- and hDPSC-originated adipocytes**. (**A**) Quantification of fluorescence emitted by lipid droplet staining in fixed cells normalised with the cell number in three separate cultures of hBMSCs at 0, 7, 10, 14, and 21 days of differentiation; the curve shows the ratio of the green staining quantification versus the total cell number. (**B**) Histogram of fluorescence emitted by lipid droplet staining in live cells at 0, 7, 10, 14, and 21 days of adipogenic differentiation of hBMSCs and graphical representation of these values. (**C**) Quantification of fluorescence emitted by lipid droplet staining in fixed cells normalised with the cell number in three separate cultures of hDPSCs at 0, 7, 10, 14, and 21 days of differentiation; the curve shows the ratio of the green staining quantification versus the total cell number. (**D**) Histogram of fluorescence emitted by lipid droplet staining in live cells at 0, 7, 10, 14, and 21 days of adipogenic differentiation of hDPSCs and graphical representation of these values. (**E**) Lipid vesicles’ number quantification normalised by the cell number from live cell lipid droplets’ staining. (**F**) Lipid vesicles’ size quantification from live cell lipid droplets’ staining. One-way ANOVA followed by Dunnett’s post hoc test was used to compare time points for each cell type. Asterisks represent statistically significant differences between different time points and T0 control (* *p* < 0.05; ** *p* < 0.01, n = 6).

Nile red and LipidSpot^TM^ tracker staining marked the lipids and lipid vesicles, respectively, in both hBMSCs and hDPSCs. Lipid droplets started to be formed at day 7 post-induction, and their number and size increased over time in the differentiating hBMSC-originated adipocytes. Lipid vesicles were well-defined, rounded, and large. Their size continuously increased during the culture period, and this was associated with a progressive increase in the fluorescence staining (Figure 2C,E, and Figure 3A,B). By contrast, in hDPSC-derived adipocytes, tiny globular small lipid vesicles started to appear around 10 days post-induction. During the 21 days of culture, the intensity of the lipid fluorescence staining increased slightly in these cells. This was concomitant with the moderate increase in the number, but not the size, of the lipid vesicles in the hDPSC-originated adipocytes (Figure 2D,F, and Figure 3C,D). Quantification of the lipid vesicles showed that their number was significantly higher in hBMSC-originated adipocytes compared to the hDPSC-derived adipocytes (Figure 3D). Similarly, the size of the vesicles was superior in hBMSC-derived adipocytes when compared to that of hDPSC-generated adipocytes (Figure 3E).

### 2.3. Commitment of hBMSCs and hDPSCs towards the Adipogenic Fate and Their Progression into Mature Adipocytes

**Figure 4:**
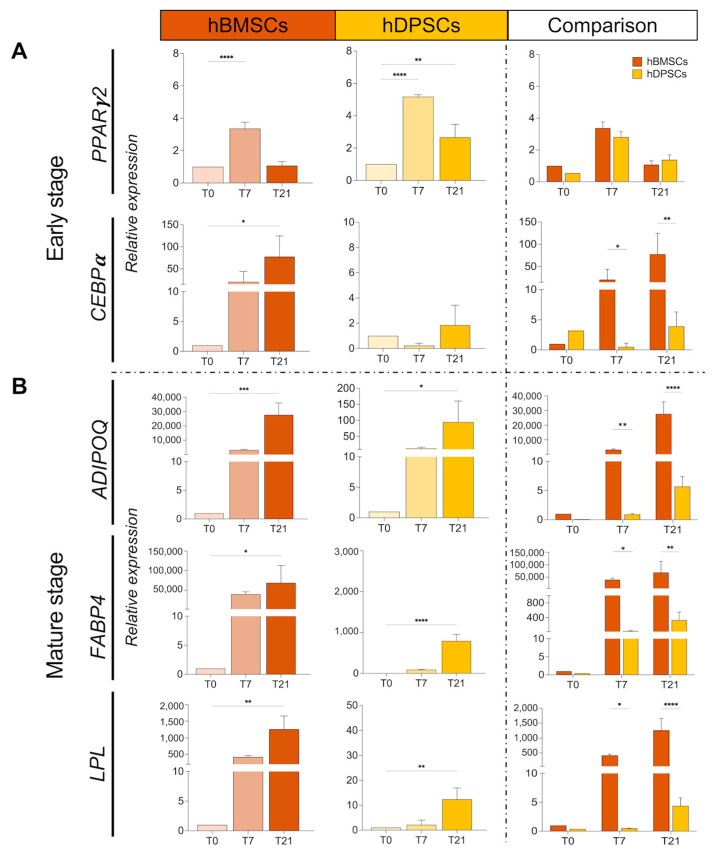
**Expression and comparison of adipogenic genes in cultured hBMSCs and hDPSCs under adipogenic conditions.** (**A**) Relative mRNA expression of the early adipogenic genes *PPARγ2* and *CEBPα* in cultured hBMSCs and hDPSCs for 0, 7, and 21 days under adipogenic conditions. (**B**) Expression of the characteristic late adipogenic genes *ADIPOQ*, *FABP4*, and *LPL* in cultured hBMSCs and hDPSCs at 0, 7, and 21 days of adipogenic induction was analysed by RT qPCR. The value of relative expression on comparison graphs is normalised to hBMSCs at T0 for each gene in statistical analysis. Data are presented as average values ± SD. One-way ANOVA followed by Dunnett’s post hoc test was used to compare time points for each cell type. Two-way ANOVA followed by Šídák post hoc test was used to compare hBMSCs and hDPSCs. Asterisks represent statistically significant differences between different time points and T0 control (* *p* < 0.05; ** *p* < 0.01; *** *p* < 0.001; **** *p* < 0.0001).

We were interested then to know the commitment of these two cell populations towards the adipocyte fate and their final differentiation into mature adipocytes. For this purpose, we examined the expression of several genes that are considered early and late markers of the adipogenic process. *PPARγ2* and *CEBPa* are two early adipocyte differentiation transcription factors [34,35]. The expression of *PPARγ2* was significantly upregulated in cultured hBMSCs only at 7 days of adipogenic induction, but in hDPSCs, its expression increased 7 and 21 days post-induction. *CEBPa* expression was rapidly and significantly upregulated in hBMSCs from 7 days post-induction, but it was only slightly increased in cultured hDPSCs (Figure 4A). Expression of *adiponectin* (*ADIPOQ*), *fatty acid binding protein 4* (*FABP4*), and *lipoprotein* (*LPL*) genes, which are involved in late stages of adipogenesis [5,36,37], showed a significant upregulation in hBMSCs at all time points of their culture (Figure 4B). By contrast, minimal upregulation of these three genes was observed in hDPSCs in comparison to hBMSCs (Figure 4B). These findings demonstrate that the high expression of the early adipogenic markers (*PPARγ2* and *CEBPa*) detected in hBMSCs at 7 days post-induction was followed up by the increased expression of late adipogenic markers (*ADIPOQ, FABP4*, and *LPL)* at 21 days (Supplementary Figure S2). Only the expression of *PPARγ2* was similarly upregulated in both hBMSCs and hDPSCs at 7 days post-induction (Figure 4A,B).

### 2.4. Comparison of the Expression of Stem Cell and Osteogenic Genes in hBMSCs and hDPSCs Cultured in Adipogenic Conditions

**Figure 5:**
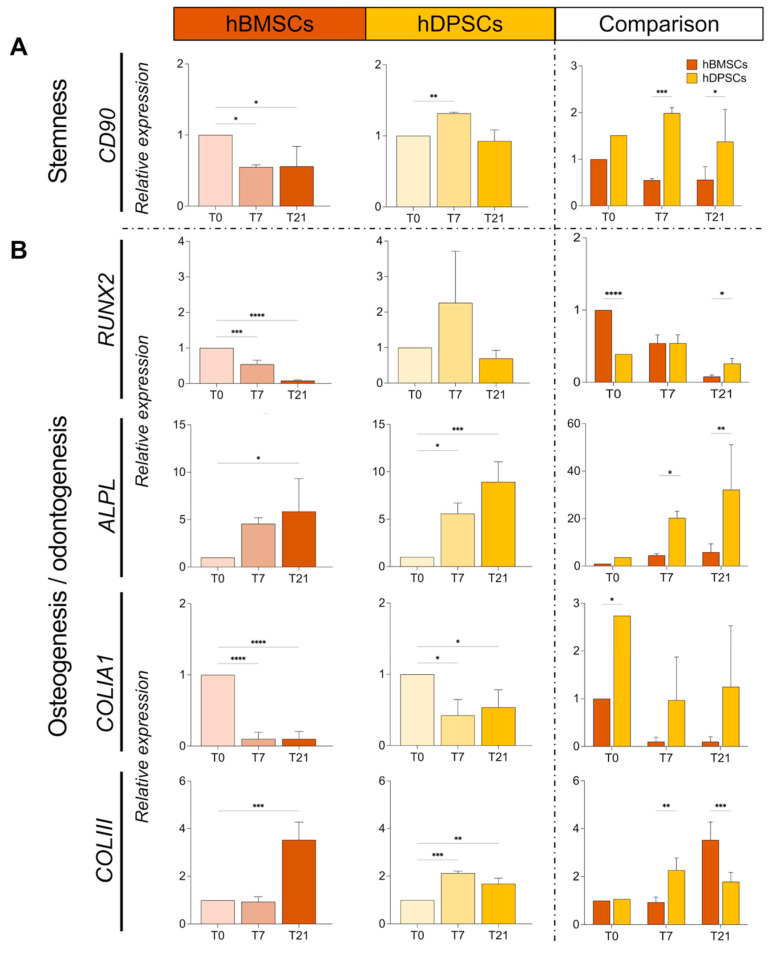
**Expression and comparison of stem cell and osteogenic genes in cultured hBMSCs and hDPSCs under adipogenic conditions**. (**A**) Relative mRNA expression of the stem cell gene *CD90* in cultured hBMSCs and hDPSCs at 0, 7, and 21 days under adipogenic conditions was analysed by RT qPCR. (**B**) Relative mRNA expression of the osteogenic genes *CD90*, *COLIII*, *ALPL*, *COLIA1*, and *RUNX2* in cultured hBMSCs and hDPSCs at 0, 7, and 21 days upon adipogenic induction was analysed by RT qPCR. The value of relative expression on comparison graphs is normalised to hBMSCs at T0 for the statistical analysis of each gene. Data are presented as average values ± SD. One-way ANOVA followed by Dunnett’s post hoc test was used to compare time points for each cell type. Two-way ANOVA followed by Šídák post hoc test was used to compare hBMSCs and hDPSCs. Asterisks represent statistically significant differences between different time points and T0 control (* *p* < 0.05; ** *p* < 0.01; *** *p* < 0.001; **** *p* < 0.0001).

We next evaluated the expression of the stem cell marker gene *CD90* [38], as well as the osteogenic and odontogenic genes *RUNX2, ALPL, COL1A1,* and *COLIII* [2,5], in hBMSCs and hDPSCs cultured in adipogenic conditions. *CD90* was dramatically decreased in hBMSCs at 7 and 21 days post-induction (Figure 5A). By contrast, the expression of *CD90* was higher in cultured hDPSCs when compared to hBMSCs (Figure 5A). *RUNX2* expression was significantly decreased in cultured hBMSCs at 7 and 21 days, whereas its expression was maintained in cultured hDPSCs (Figure 5B). While expression of *COLIII, COLIA1,* and *ALPL* was upregulated or maintained in cultured hDPSCs for 21 days, their expression was inferior in hBMSCs at all time points of their culture (Figure 5B, Supplementary Figure S2).

### 2.5. Comparison of the Expression of WNT and SWAT Cell-Related Genes in hBMSCs and hDPSCs Cultured in Adipogenic Conditions

**Figure 6:**
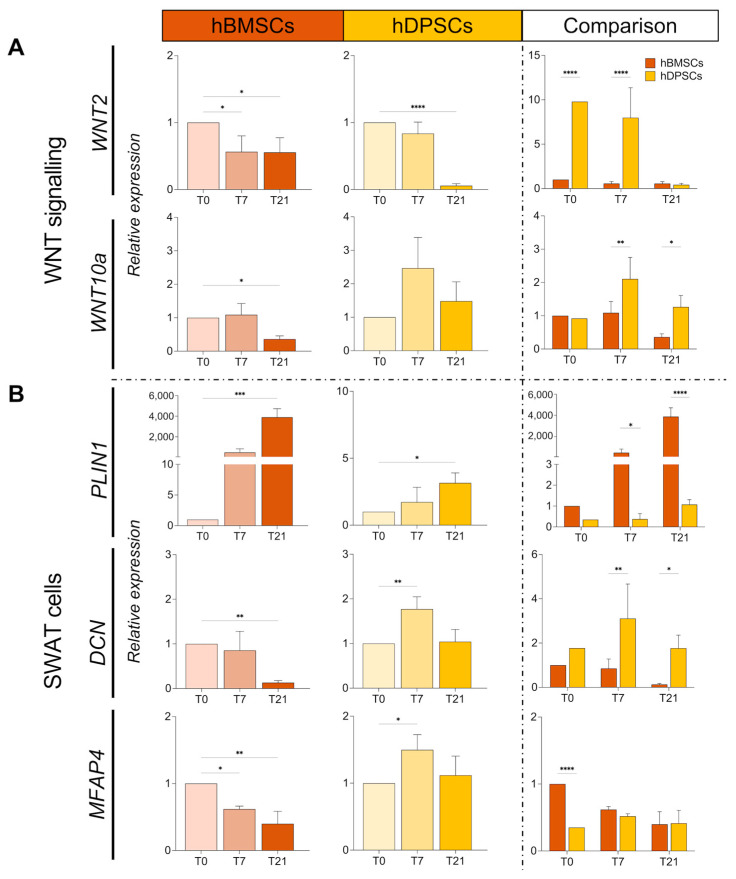
**Expression and comparison of WNT and SWAT cell genes in hBMSCs and hDPSCs cultured under adipogenic conditions.** (**A**) Relative mRNA expression of the *WNT2* and *WNT10A* genes in cultured hBMSCs and hDPSCs at 0, 7, and 21 days under adipogenic conditions was analysed by RT qPCR. (**B**) Relative mRNA expression of the *PLIN1*, *DCN*, and *MFAP4* genes in cultured hBMSCs and hDPSCs at 0, 7, and 21 days of adipogenic induction was analysed by RT qPCR. The value of relative expression on comparison graphs is normalised to hBMSCs at T0 for each gene. Statistical analysis data are presented as average values ± SD. One-way ANOVA followed by Dunnett’s post hoc test was used to compare time points for each cell type. Two-way ANOVA followed by Šídák post hoc test was used for comparison between hBMSCs and hDPSCs. Asterisks represent statistically significant differences between different time points and T0 control (* *p* < 0.05; ** *p* < 0.01; *** *p* < 0.001; **** *p* < 0.0001).

Next, we analysed the expression of *WNT10a* and *WNT2*, as well as the expression of periplin 1 (*PLIN1*), decorin (*DCN*), and microfibril-associated glycoprotein 4 (*MFAP4*), which are genes associated with the multipotent profile of SWAT cells [39]. Expression of the *WNT10a* and *WNT2* genes was considerably decreased in hBMSCs cultured for 21 days in adipogenic conditions, while their expression was significantly higher in hDPSCs at all time points of culture (Figure 6A). While *PLIN1* expression was gradually upregulated in hBMSCs upon adipogenic induction, its expression was slightly modified in cultured hDPSCs (Figure 6B). By contrast, *DCN* and *MFAP4* expression was significantly downregulated in cultured hBMSCs, while the expression of *DCN*, but not of *MFAP4*, was drastically upregulated in hDPSCs over the adipogenic culture period (Figure 6B, Supplementary Figure S2). 

### 2.6. Expression of Notch Pathway Genes in hBMSCs and hDPSCs Cultured in Adipogenic Conditions

**Figure 7:**
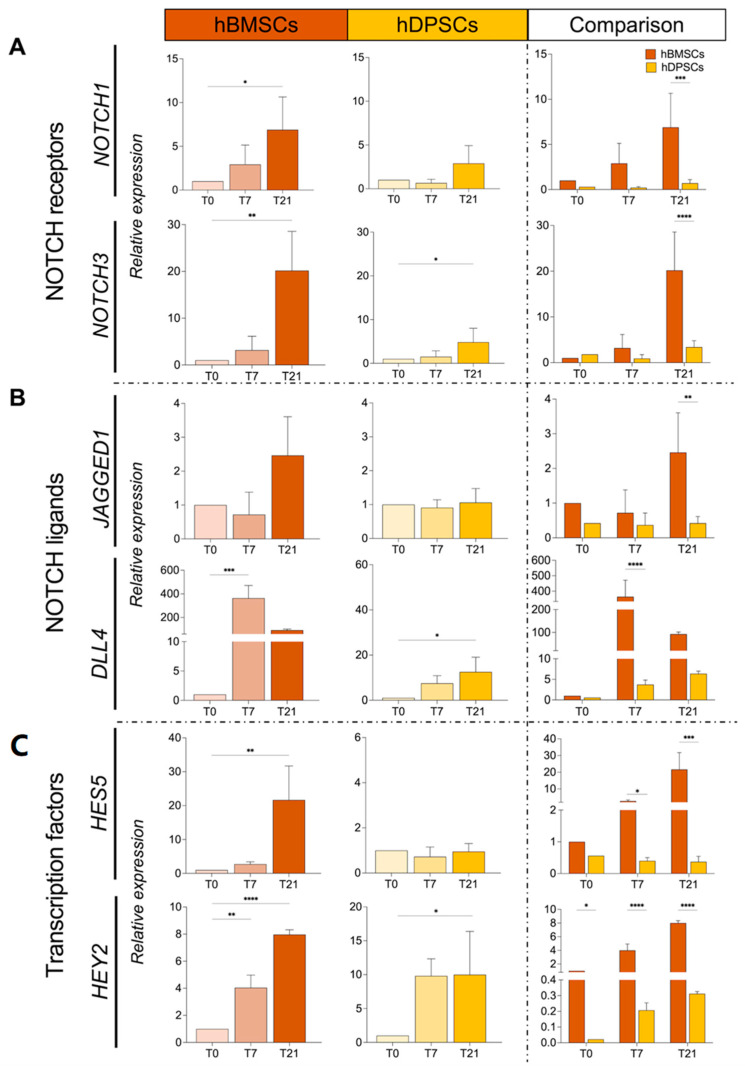
**Expression and comparison of NOTCH pathway genes in hBMSCs and hDPSCs cultured in adipogenic conditions**. (**A**) Relative mRNA expression of *NOTCH1* and *NOTCH3* in cultured hBMSCs and hDPSCs at 0, 7, and 21 days of adipogenic induction. (**B**) Relative mRNA expression of *JAGGED1* and *DELTA-LIKE4* (*DLL4*) in cultured hBMSCs and hDPSCs at 0, 7, and 21 days of adipogenic induction. (**C**) Relative mRNA expression of the Notch pathway transcription factors *HES5* and *HEY2* in cultured hBMSCs and hDPSCs at 0, 7, and 21 days of adipogenic induction. The value of relative expression on comparison graphs is normalised to hBMSCs at T0 for each gene. Statistical analysis data are presented as average values ± SD. One-way ANOVA followed by Dunnett’s post hoc test was used to compare time points for each cell type. Two-way ANOVA followed by Šídák post hoc test was used to compare hBMSCs and hDPSCs. Asterisks represent statistically significant differences between different time points and T0 control (* *p* < 0.05; ** *p* < 0.01; *** *p* < 0.001; **** *p* < 0.0001).

We further investigated and compared the expression of genes involved in the NOTCH signalling pathway in hBMSCs and hDPSCs cultured in adipogenic media. *NOTCH1* and *NOTCH3* expression was gradually increased in hBMSCs upon 21 days of adipogenic induction, while, in hDPSCs, these two genes were minimally expressed at all time points (Figure 7A). Expression of *JAGGED1* and *DELTA-LIKE4 (DLL4),* and the Notch downstream genes *HES5* and *HEY2*, was significantly upregulated in hBMSCs after adipogenic induction, while expression of these genes was slightly affected in cultured hDPSCs *(*Figure 7B,C). No major changes in the expression of *NOTCH2* and *HES1* were observed in both cell populations under adipogenic conditions, while increased *HEY1* expression was seen only 7 days post-induction in hBMSCs but not in hDPSCs (Supplementary Figures S1 and S2).

## 3. Discussion

Although bone marrow stem cells (BMSCs) are the gold standard for mesenchymal stem cell (MSC) plasticity, regulation of their commitment to the adipogenic lineage is a complex process that is not yet fully understood [6,14,16,40]. This process likely involves molecules such as steroid hormones, secreted cytokines, and transcription factors like CCAAT/enhancer-binding proteins (C/EBPs) and peroxisome proliferator-activated receptor γ2 (PPARγ2) [35,37,41]. Following cell commitment, a cascade of differentiation events, controlled by time-dependent molecular mechanisms, results in the generation of mature adipocytes [6,42,43]. Hence, the progression of the adipogenic lineage is defined by specific gene expression patterns [5,39,42,43]. The early differentiation stage (preadipocyte stage) is characterised by an increase in *PPARγ* and *C-EBPa* expression, while the rise of adiponectin complement-related protein (*ADIPOQ)* and fatty-acid-binding protein 4 (*FABP4)* marks the later stages [5,34]. MSCs isolated from other tissues than bone marrow follow the same molecular route of adipogenic differentiation [6,34,43], yet the extent of adipogenic potential significantly varies between these various sources [7,23,24,44]. Therefore, to analyse the adipogenic differentiation potential of human-derived BMSCs (hBMSCs) and human-derived dental pulp stem cells (hDPSCs), we performed a comprehensive, quantitative, and comparative study in vitro.

Previous studies have shown that hBMSCs and hDPSCs have the potential to differentiate into many cell lines, including osteoblasts, chondroblasts, neuronal cells, myogenic cells, and adipocytes [7,16,17,45,46,47]. Here, we show that hDPSCs display distinctive morphological and molecular specificities upon adipogenic differentiation compared to hBMSCs. The onset of adipogenesis in hDPSCs was delayed, and the extent of adipogenic differentiation is significantly reduced when compared to hBMSCs. The presence of early markers of adipogenesis (*PPARγ, C-EBPa*) indicates that hDPSCs can commit to the adipocyte lineage, but they lack the capacity to successfully generate mature adipocytes. This is further supported by the poor quality and small number of lipid vacuoles observed in the hDPSC-derived adipocytes. Indeed, hDPSC-derived adipocytes exhibited a granular appearance due to the small size of the lipid vesicles, in contrast to the hBMSC-derived adipocytes, which showed an increased number and size of lipid vesicles [48,49].

A balance between the osteogenic and adipogenic differentiation potential exists in vivo [6,39,50]. The commitment of MSCs towards one of these two specific fates is in part regulated by antagonistic interactions between runt-related transcription factor 2 (RUNX2) and PPARγ, which are key regulators of osteogenesis and adipogenesis, respectively [2,4,5,35]. Our in vitro results show increased *RUNX2* expression in hDPSCs after the first week of their adipogenic induction, which likely inhibits adipogenic differentiation despite the increased expression of *PPARγ*. This is further confirmed by the high expression levels of collagen type I alpha 1 (*COL1A1*), bone sialoprotein *(BSP)*, alkaline phosphatase (*ALPL),* and *RUNX2*, which were maintained and not decreased throughout hDPSCs’ adipogenic induction, thus indicating the persistence of their osteogenic/dentinogenic differentiation potential.

Recently published studies have identified two distinct developmental trajectories for the differentiation of adipocyte progenitors: the first one generates mature adipocytes, while the other one that is regulated by Wnt signalling allows them to remain in their progenitor state [9,39]. This cell fate determination depends on the culture media that can either generate mature adipocytes or maintain progenitors’ multipotency. These latter cells are named structural Wnt-regulated adipose-tissue-resident (SWAT) cells and express genes encoding osteoblast-specific extracellular matrix proteins, which inhibit adipogenic differentiation while maintaining their progenitor status [8,36]. Maintenance of osteoblastic genes in hDPSCs such as *COL1A1, BSP, ALPL,* and *RUNX2* upon adipogenic induction indicates less commitment towards adipocyte differentiation and maturation when compared to hBMSCs. This is further supported by the concomitant increased expression of *decorin* (*DCN)* and decreased expression of *perilipin 1* (*PLIN1)*, which are markers of SWAT cells [31,46], in hDPSCs cultured in adipogenic conditions. By contrast, hBMSCs cultured in adipogenic medium were entirely committed to becoming adipocytes and showed a limited expression of osteoblastic genes and reversed hDPSC expression patterns for *PLIN1* and *DCN*. The co-expression of *ADIPOQ, LPL,* and *PLIN1* and the decreasing expression of *DCN* and *MFAP4* in hBMSCs are consistent with the adipogenic commitment of hBMSCs and the loss of SWAT molecular signature [9,39].

Our findings indicate a correlation between the late differentiation stages of adipogenesis in hBMSCs and elevated expression of the Notch pathway components, including receptors (*NOTCH1* and *NOTCH3)*, ligands (*JAGGED1* and *DELTA-LIKE4)*, and downstream genes (*HES5* and *HEY2)*. Conversely, the modest upregulation of these genes is associated with the SWAT-like phenotype found in hDPSCs. The Notch signalling pathway regulates cell fate choices during embryogenesis and regeneration of most tissues and organs [51,52]. While it is largely accepted that the Notch pathway acts as a negative regulator of adipogenesis, its role in this process still remains controversial and unclear [53]. Previous studies have shown that the blockage of Notch signalling by γ-secretase inhibitors promotes the adipogenic differentiation of MSCs and hBMSCs [54,55], while Notch signalling activation inhibits hBMSC differentiation into adipocytes and decreases the expression of adipogenic genes, including *PPAR*γ and *ADIPONECTIN* [56]. A recent study on human adipose-derived stem cells (hADSCs) has shown that Notch3 is involved in early adipogenic differentiation, prior to the formation of lipid vesicles, while Notch1 functions at later differentiation stages [53]. In vitro studies using 3T3-L1 and C3H10T1/2 cells have shown that a lack of Notch1 downregulates the expression of genes coding for fatty-acid-activated transcription factors, such as *PPARγ* [55,57], while another study has demonstrated that the overexpression of the full-length Notch1 upregulates *PPARγ* expression in 3T3-L1 cells [58]. Although the present study reveals the expression of individual NOTCH receptors, ligands, and downstream regulators in hBMSCs and hDPSCs cultured in adipogenic conditions, our results are insufficient to explain the precise function of Notch signalling during adipogenesis.

## 4. Materials and Methods

### 4.1. Collection of Human Teeth and Dental Pulp Cells

All experiments were performed at the Centre of Dental Medicine of the University of Zurich and approved by the Ethics Committee of the Canton of Zurich (reference number 2012-0588, renewal of the canton notification and its final decision: received on 7 November 2023, ref. number: A230123-00). Healthy human teeth (wisdom teeth) were obtained from anonymous patients between 18 and 35 years of age and after their written informed consent. The teeth were extracted by surgeons and dentists at the Clinic of Oral Surgery Department, and all procedures were implemented following the current guidelines [17,23,59]. Then, human dental pulp stem cells (hDPSCs) were isolated from the dental pulp of extracted wisdom teeth of healthy patients as previously described [17,23,59]. For this study, a total of 30 fresh dental pulps were extracted from 15 teeth of separate individuals following the same criteria previously described [17,23,59]. Briefly, the dental pulp tissues were gently removed, minced, rinsed with PBS, and finally were enzymatically digested for 1h at 37 °C in a solution of collagenase (3 mg/mL; Life Technologies Europe BV, Zug, Switzerland) and dispase (4 mg/mL; Sigma-Aldrich Chemie GmbH, Buchs, Switzerland).

Human bone marrow stem cells (hBMSCs) were obtained from Lonza (Basel, Switzerland). These cells were isolated from the bone marrow of posterior iliac crests of adult healthy individuals. hBMSCs were cryopreserved after their second culture passage. A certificate reporting on the health status of the purchased cells was provided.

hDPSCs and hBMSCs were both tested and found negative for HIV-1, hepatitis B, hepatitis C, mycoplasma, bacteria, yeast, and fungi.

### 4.2. Cell Cultures and Differentiation Assays

hDPSCs and hBMSCs were initially seeded at a density of 5 × 10^3^ cell/cm^2^ in T25 flasks with medium containing modified minimum essential medium α (MEMα) without ribonucleosides or deoxyribonucleosides and phenol red (Sigma-Aldrich Chemie GmbH, Buchs, Switzerland), supplemented with 10% heat-inactivated foetal bovine serum (FBS) (Bioswisstech, Schaffhausen, Switzerland), 1% penicillin/streptomycin (P/S) (Sigma-Aldrich Chemie GmbH, Buchs, Switzerland), 1% L-glutamine (Sigma-Aldrich Chemie GmbH, Buchs, Switzerland), and 0.5 μg/mL fungizone (Life Technologies Europe BV, Zug, Switzerland) after washing away the enzyme solution. The culture medium was changed every 3 days. Cells were passaged at 80–90% confluence and expanded in the same growth medium in T75 culture flasks. Cells were used up to a maximum passage of 15 (P15). Morphological changes were not observed during all these passages.

For differentiation assays, cells from T75 flasks were washed with PBS before trypsin (0.05% EDTA) was added for 3 min at 37  °C for their detachment, once a confluence of 80–90% was reached. Trypsin was blocked by addition of 5 volumes of the culture medium with 10% FBS. After centrifugation, 5 × 10^4^ cells per well were seeded onto 24-well plates (Sarsted, Switzerland) or in μ-slide 4-well uncoated plates (ref: 80,821, IBIDI GmbH, Gräfelfing, Germany) for fluorescence stainings and microscopic analyses, while for gene expression analysis, 2 × 10^5^ cells were seeded onto 6-well plates (Sarsted, Switzerland) in triplicates.

The adipogenic differentiation medium (AMd) consisted of Dulbecco’s modified Eagle medium/nutrient mixture F-12 (DMEM-12, ThermoFisher/Life Technologies, Zug, Switzerland), 100 μM sodium pyruvate, and 1% P/S, supplemented with dexamethasone (1 μM) (Sigma-Aldrich Chemie GmbH, Buchs, Switzerland), 3-isobutyl-1-methylxanthine (IBMX; 0.5 mM), indomethacin (200 μM) (Sigma-Aldrich Chemie GmbH, Buchs, Switzerland), insulin (10 μM) (Sigma-Aldrich Chemie GmbH, Buchs, Switzerland), and amphotericin B (0.25 μg/μL) (Life Technologies Europe BV, Zug, Switzerland) [7,23]. The medium was changed every 3 days and cells were cultured for up to 21 days in AMd. Briefly, cells were collected from the 6-well plates on days 0 (the plating day), 7, and 21 and used for RNA extraction. Cells cultured on 24-well plates or in μ-slide 4-well-uncoated plates were cultured, then stained and examined under a bright-field microscope after 0, 7, 14, and 21 days (Figure 1).

### 4.3. Staining

Nile red staining: Nile Red (9-diethylamino-5H-benzo[alpha]phenoxazine-5-one) (Sigma-Aldrich Chemie GmbH, Buchs, Switzerland) is a lipophilic vital stain for the detection of intracellular lipid droplets with a yellow-gold fluorescence colour [60]. μ-Slide 4-well uncoated plates were used to culture cells in adipogenic medium, supplemented with the Nile red dye diluted to 1:1000 in 1 mL of the culture medium. The Nile red incubation period was 1h at 37 °C in an atmosphere of 5% CO_2_. Lipid droplets within the cells were imaged by epifluorescence microscopy (Leica DFC7000T, CCR: Ex = 562:598 nm, Em = 610:710 nm) [60,61].

LipidSpot^TM^ 488 lipid droplet staining: LipidSpot^TM^ 488 dyes are fluorogenic neutral stains for the detection of lipid droplets within living or fixed cells (ThermoFisher/Life Technologies, Zug, Switzerland). We performed the staining in fixed cells that were cultured in 24-well plates. Cells were fixed with 4% PFA for 30 min and washed with deionised water before staining with the LipidSpot^TM^ 488 dyes that were diluted 1:100 in PBS. The 24-well plates were then incubated for 45 min at 37 °C, washed with PBS, followed by a nucleic acid staining with 4′,6-diamidino-2-phenylindole, dilactate (DAPI; ThermoFisher Scientific, Reinach, Switzerland) (1:100 dilution in PBS), and finally fluorescence images were captured by confocal microscopy in the appropriate detection channels that show the lipid droplets in green and cell nuclei in blue [60,61].

### 4.4. Imaging

Confocal high-speed multispectral spinning-disk microscopy: We used a multispectral spinning-disk confocal microscope on the Olympus IXplore SpinSR10 super resolution imaging system (with a YOKOGAWA CSU-W1 spinning disk, which reduces phototoxicity and bleaching, and the Olympus Z-drift compensator (IX3-ZDC2) which maintains focus). It is equipped with two sCMOS cameras, which allow fast and sensitive simultaneous acquisition of two-colour-labelled (live) samples, and with a motorised xyz stage (IX3-SSU), Olympus real-time controller (U-RTCE), and the Olympus Z-drift compensator (IX3-ZDC2). The objective used was a UPLSAPO UPlan S Apo, magnification 30×, NA: 1.05, immersion in silicon oil. The original photos acquired from each fluorescence channel were converted to colour, with contrast and brightness adjustments, and unsharp masking filters were used to create the figure in FIJI (free version: 2.9.0).

Light wide-field microscopy imaging: Sections were imaged using the wide-field DM6000B Leica microscope supplemented with the DFC420C Leica camera and the Leica LAS X 4.4 Falcon Lightning software (Version 4.4.0.24861, Center for Microscopy and Image Analysis, Zurich, Switzerland). All cultured wells with living or fixed cells were imaged with 20× and 40× objectives (Leica HC PL APO CS2, 0.75 Dry) and single images were merged together using the LAS X software (Leica) and FIJI.

### 4.5. RNA Extraction, Reverse Transcription, and Quantitative Real-Time PCR (qRT-PCR)

Total RNA was extracted from cells cultured in 6-well plates using TRIzol^TM^ reagent (Ref No. 15596026; ThermoFisher/Life Technologies, Switzerland) according to the manufacturer’s instructions. Briefly, 500 µL of TRIzol^TM^ was added to the 6-well culture plates after the media had been taken away and cells scratched with a micropipette point. The lysate was incubated with 0.1 mL of chloroform for 3 min at room temperature, followed by centrifugation at 12,000× *g* for 15 min at 4 °C. The aqueous phase containing the RNA was separated and placed in a spin column with gDNA eliminator and DNAse I (Sigma-Aldrich Chemie GmbH, Buchs, Switzerland). A NanoDrop (Q6000; QUAWELL Technology Limited, LabGene Scientific, Châtel-Saint-Denis, Switzerland) was used to measure the total RNA concentration. The concentration was then narrowed to 250 ng/µL for all samples. Reverse transcription of the isolated RNA was performed using the iScript™ cDNA synthesis kit (Cat. No. 1706691; Bio-Rad Laboratories AG, Hercules, CA, USA) according to the manufacturer’s instructions. Relative mRNA expression levels were evaluated using the SYBR Green method. The quantitative 3-step real-time PCR was performed by the Eco Real-Time PCR system (96-well 0,1 mL Block, A28131, Applied Biosystems, ThermoFisher, Switzerland). Amplification reactions were carried out using the LightCycler^®^ 480 SYBR Green I Master (Roche Life Science, Switzerland), as previously described [7,23,59]. Primer sequences for the genes *CD90, WNT2*, *WNT10A*, *RUNX-2*, *ALPL*, *COL1A1*, *COL3*, *CEBPα*, *PPARγ2*, *ADIPQ*, *FABP4*, *LPL*, *PLIN1*, *DCN*, *MFAP4*, *NOTCH1*, *NOTCH2*, *NOTCH3*, *JAGGED2*, *HES1*, *HES5*, *HEY1*, *HEY2*, and *DLL4* were bought from the manufacturer (Microsynth AG, Balgach, Switzerland). The sequences of the primers are supplied in Table 1.

In all samples, GAPDH served as the endogenous control (housekeeping gene). The thermocycling conditions were: 95 °C for 10 min, followed by 40 cycles of 95 °C for 15 s, 55 °C for 30 s, and 60 °C for 1 min. Melt curve analysis was performed at 95 °C for 15 s, 55 °C for 15 s, and 95 °C for 15 s. Expression levels were calculated by the comparative ΔΔCt method (2^−ΔΔCt^ formula), after being normalised to the Ct value of the GAPDH housekeeping gene. The expression of each gene was presented as relative to the reference gene and normalised for the expression of the gene at day 0 in each cell lineage. All values of relative expression for each gene in each cell type at 0, 7, 14, and 21 days of differentiation were plotted in a heatmap, which displayed previously clustered data obtained from the qRT-PCR on the web server at http://www.heatmapper.ca (accessed on 29 January 2024).

### 4.6. Quantification of Staining and Statistical Analysis

Yellow- or green-coloured lipid droplets were counted in FIJI. Quantitative analyses were performed based on values calculated in FIJI. These values were obtained from histograms and segmentation of the fluorescence, intensity measurements, measurements by manual counter, and particle analysis performed in FIJI. The fluorescence mean values of the 2 staining histograms extracted from FIJI were calculated in ratio with the cell number in each image, referring to the DAPI nuclear staining, and thanks to the particle analyser. Secondly, the number of lipid particles and the areas covered by these particles at each time point were normalised according to cell number and particle average size. For each staining analysis and microscopy technique, three measurements from each experimental and biological triplicate were obtained and quantified in FIJI. RT-qPCR analysis was also performed in 3 separated biological samples and in experimental triplicates.

Statistical analysis was performed using the pairwise ANOVA test and Dunnett multicomparison post hoc test for RT-qPCR and vesicle quantification for each cell type. A two-way ANOVA and Šídák post hoc test were used for the comparison between hDPSCs and hBMSCs. The statistical significance level was considered to be *p*  <  0.05. The data were analysed using PRISM 6.0 (GraphPad, La Jolla, CA, USA).

## 5. Conclusions

**Figure 8:**
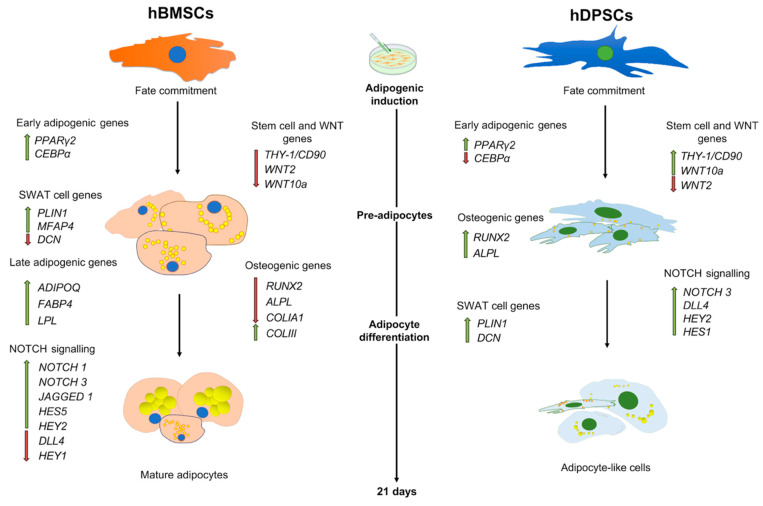
**Schematic representation of the differentiation of cultured hBMSCs and hDPSCs under adipogenic conditions and the expression of various genes during the differentiation process.** Cultured hBMSCs form numerous large lipid droplets (yellow colour). Upregulation (green arrows) of adipogenic genes and concomitant downregulation (red arrows) of WNT, stem cell, and osteogenic genes indicate their unreserved commitment towards the adipogenic fate. By contrast, hDPSCs form tiny and less numerous lipid vesicles when compared to hBMSCs and demonstrate a less evident adipocyte morphology. Furthermore, hDPSCs keep high expression of most WNT, stem cell, and osteogenic genes, which indicates their partial commitment towards the adipogenic fate.

In conclusion, our results show that hDPSCs have a limited adipogenic differentiation potential when compared to hBMSCs. Furthermore, hDPSCs maintain the expression levels of osteogenic/dentinogenic genes even under adipogenic culture conditions (Figure 8). One of the key properties of MSCs is their ability to differentiate into mesodermal cell lineages, including adipocytes [1,2,10]. The limited adipogenic differentiation potential of hDPSCs in vitro when compared to BMSCs suggests that MSCs from different organs exhibit functional differences. These differences may stem from inadequate induction conditions in vitro, or they could reflect intrinsic biological distinctions between MSC populations. For example, unlike bone marrow, dental pulp is devoid of adipocytes in vivo and does not generate them when transplanted into mice [17]. While our study does not provide a definitive answer to this question, it underscores the need for further experiments to classify these functional differences, which could have a direct impact on the outcome of regenerative therapies.

## Figures and Tables

**Table 1:** Primers used in the study.

Gene	Accession No.	Forward Primer 5′–3′	Reverse Primer 5′–3′
*GAPDH*	NM_002046.5	AGGGCTGCTTTTAACTCTGGT	CCCCACTTGATTTTGGAGGGA
*CD90*	NM_006288.3	GAAGGTCCTCTACTTATCCGCC	TGATGCCCTCACACTTGACCAG
*WNT2*	NM_003391.3	AGGATGCCAGAGCCCTGATGAA	AGCCAGCATGTCCTGAGAGTAC
*WNT10B*	NM_003394.2	CTCGGGATTTCTTGGATTCCAGG	GCCATGACACTTGCATTTCCGC
*RUNX2*	NG_008020.1	GCCAGGGTCTAGGAGTTGTT	ACCCACCACCCTATTTCCTG
*COL1A1*	NM_000088.1	CCAGAAGAACTGGTACATCAGCAA	CGCCATACTCGAACTGGAATC
*COL3*	NM_000090	TGGTCTGCAAGGAATGCCTGGA	TCTTTCCCTGGGACACCATCAG
*PPAR-* *γ* *2*	NM_138712.3	GAACGACCAAGTAACTCTCC	CGCAGGCTCTTTAGAAACTCC
*ADIPOQ*	NM_004797	CAGGCCGTGATGGCAGAGATG	GGTTTCACCGATGTCTCCCTTAG
*FABP4*	NM_001442	ACGAGAGGATGATAAACTGGTGG	GCGAACTTCAGTCCAGGTCAAC
*LPL*	NM_000237.2	ACGGCATGTGAATTCTGTGA	GGATGTGCTATTTGGCCACT
*C/EBP* *α*	NM_024988.1	GGAGGAGACAAACTTAACTCTGG	ACACCCTCGCTCCCGCCGTT
*PLIN1*	NM_002666.1	GCGGAATTTGCTGCCAACACTC	AGACTTCTGGGCTTGCTGGTGT
*ALPL*	NM_000478.1	GCTGTAAGGACATCGCCTACCA	CCTGGCTTTCTCGTCACTCTCA
*DCN*	NM_001920.1	GCTCTCCTACATCCGCATTGCT	GTCCTTTCAGGCTAGCTGCATC
*MFAP4*	NM_002404.2	GGCTCAGTAAGTTTCTTCCGCG	CCAAGTCCACTCGCAGCTCATA
*NOTCH1*	NM_017617.2	GGTGAACTGCTCTGAGGAGATC	GGATTGCAGTCGTCCACGTTGA
*NOTCH2*	NM_024408.1	TTCTGGAAATTGACAACCGC	CAAGAGGGTATGACAGGGTCC
*NOTCH3*	NM_000435.1	GGGACTACAAGAAGAGGAGC	GGAATTCAGCTACACAGGGA
*JAGGED1*	NM_000214	CGACCCCCTGTGAAGTGATT	ACTCTTGCACTTCCCGTGAG
*HES1*	NM_005524.1	GCTCTGAAGAAAGATAGCTCGC	GTTCCGGAGGTGCTTCACT
*HEY1*	NM_012258.1	TAATTGAGAAGCGCCGACGA	GCTTAGCAGATCCTTGCTCCA
*HEY2*	NM_012259.1	TGAGAAGACTTGTGCCAACTGCT	CCCTGTTGCCTGAAGCATCTTC
*HES5*	NM_001010926.1	TCCTGGAGATGGCTGTCAGCTA	CGTGGAGCGTCAGGAACTGCA
*DLL4*	NM_019074.1	CCAGGGACTCCATGTACCAG	CCTGCCTTATACCTCCGTGG

**Abbreviations: *GAPDH***, glyceraldehyde-3-phosphate dehydrogenase; ***CD90-THY-1***, cluster of differentiation 90–THY-monocyte differentiation antigen 1; ***WNT2***, Wingless-type MMTV integration site family, member 2; ***WNT10A***, wingless-type mmtv integration site family, member 10a; ***RUNX2***, runt-related transcription factor 2; ***COL1A1****,* collagen type I alpha 1 chain; ***COL3***, collagen type III; ***PPAR-γ2***, peroxisome proliferator-activated receptor; ***ADIPOQ***, adiponectin or adipocyte complement-related protein; ***FABP4***, fatty-acid-binding protein 4; ***LPL***, lipoprotein lipase; ***CEBPα***, ccaat enhancer binding protein alpha; ***PLIN1***, perilipin 1; ***ALPL***, alkaline phosphatase; ***DCN***, decorin; ***MFAP4***, microfibril-associated protein 4; ***NOTCH1***, notch receptor 1; ***NOTCH2***, notch receptor 2; ***NOTCH3***, notch receptor 3; ***JAGGED1***, jagged canonical notch ligand 1; ***HES1***, hes family bhlh transcription factor 1; ***HEY1***, hairy/enhancer-of-split-related protein 1; ***HEY2***, hairy/enhancer-of-split protein 2; ***HES5***, hes family bhlh transcription factor 5; ***DLL4***, delta-like canonical notch ligand 4.

## Data Availability

Data is contained within the article or Supplementary Material.

## Supplementary Materials

The following supporting information can be downloaded at: https://www.mdpi.com/article/10.3390/ijms252011105/s1.
