# Supplementary material for: Limited Adipogenic Differentiation Potential of Human Dental Pulp Stem Cells Compared to Human Bone Marrow Stem Cells
Source: Int J Mol Sci. 2024 Oct 16;25(20):11105. doi: 10.3390/ijms252011105 (PMC11508566; doi:10.3390/ijms252011105)
Supplement: Supplementary file 1 [file ijms-25-11105-s001.zip › ijms-3256867-supplementary.pdf]

Supplemental Figure S1

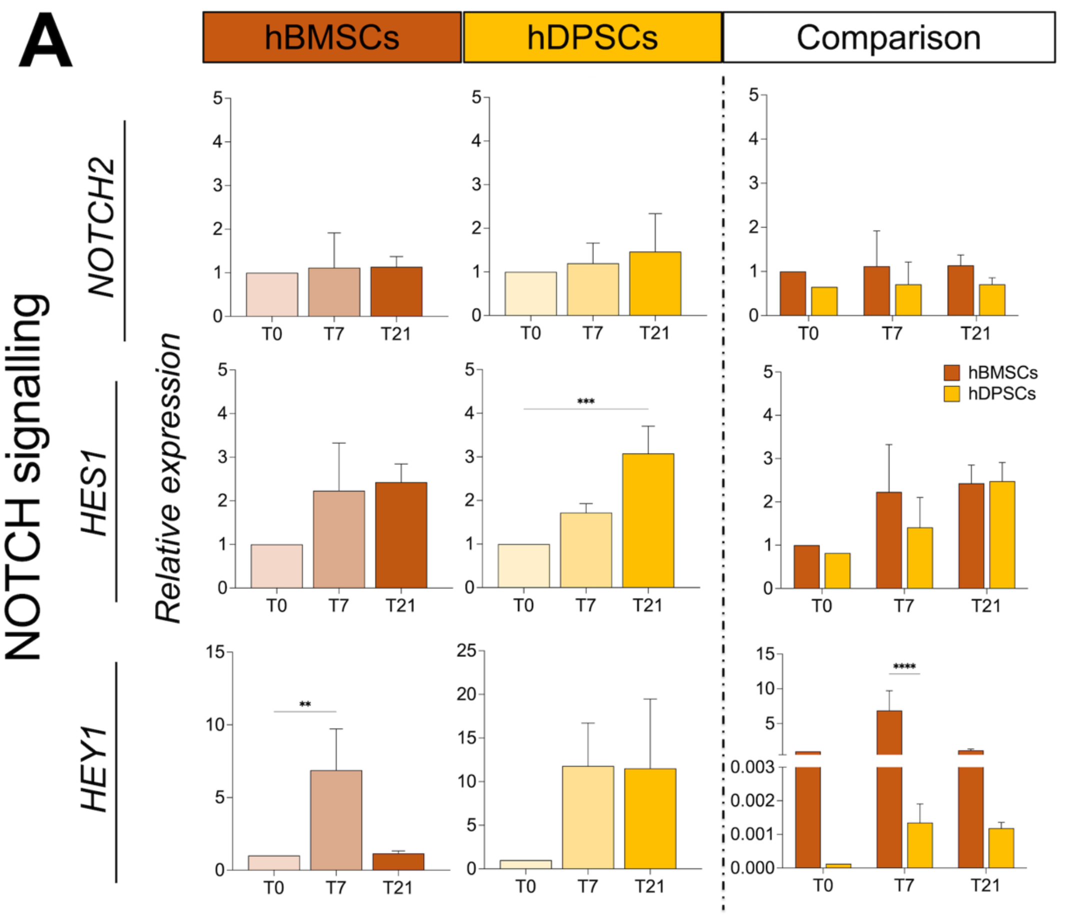

Supplemental Figure S1. ***Expression and comparison of NOTCH2, HES1* and *HEY1 in hBMSCs and hDPSCs cultured in adipogenic conditions.***

A. Relative mRNA expression of *NOTCH2*, *HES1* and *HEY1* in cultured hBMSCs and hDPSCs at 0, 7, and 21 days of adipogenic induction. The value of relative expression on comparison graphs is normalised to hBMSCs at T0 for each gene. Statistical analysis data are presented as average values ± SD. One-way ANOVA followed by Dunnett's post-hoc was used to compare time points for each cell type. Two-way ANOVA followed by Šídák post-hoc was used to compare hBMSCs and hDPSCs. Asterisks represent statistically significant differences between different time points and its T0 control (*$P < 0.05$; **$P < 0.01$; ***$P < 0.001$; ****$P < 0.0001$).

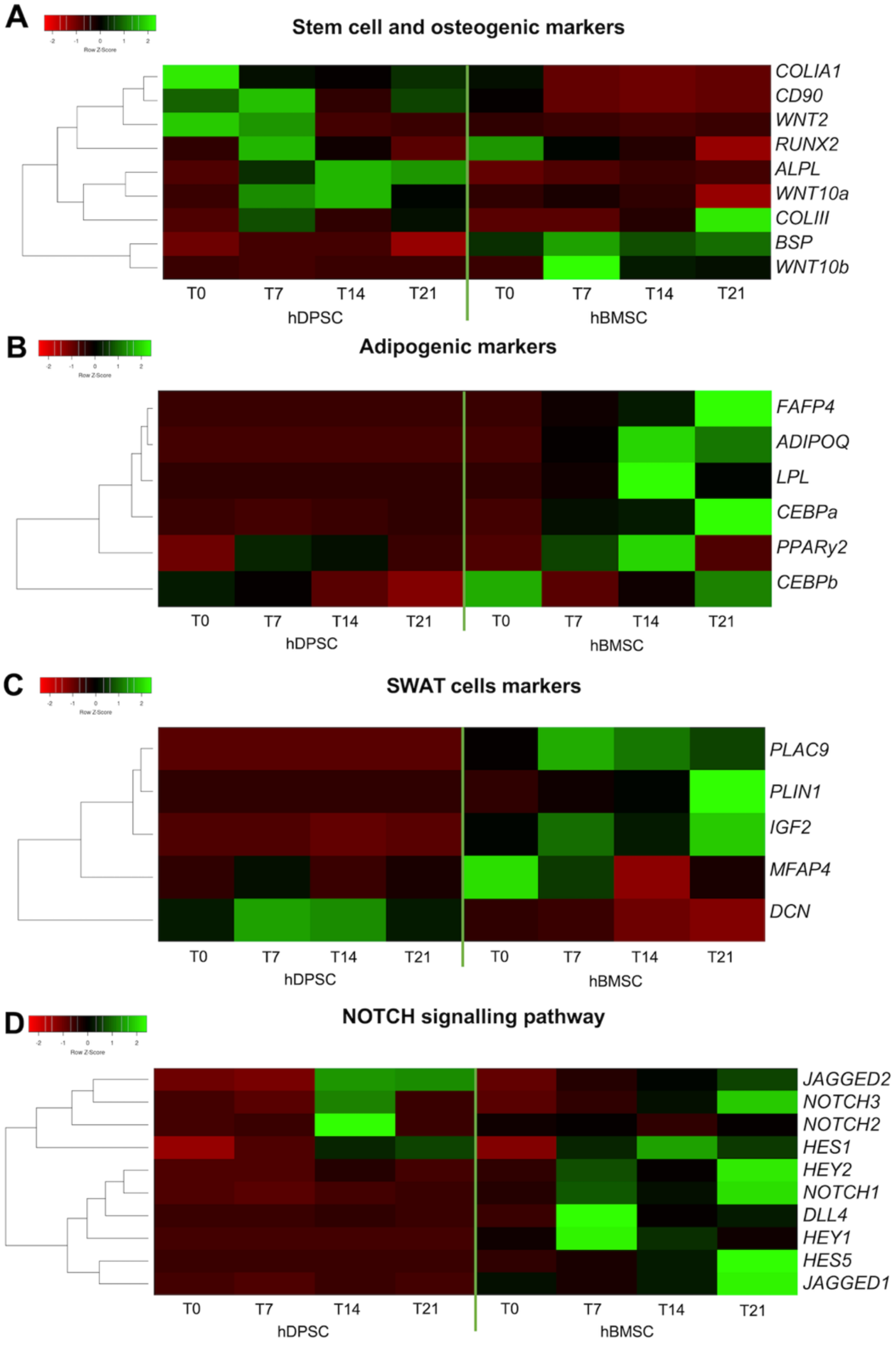

Supplemental Figure S2

Supplemental Figure S2. ***Heat Map of the cluster genes.***

A. Heat map of clustered genes analysis of stem cell and differentiation genes; **B**. Adipogenic genes; **C.** SWAT cells genes, and **D.** NOTCH signalling pathway genes. Their expression is represented at all time points based on the spearman correlation index when comparing the expression to hBMSCs at T0 for each gene. A green colour indicates a higher expression of the gene and red colour indicates a lower expression.
